# 2,2′,2′′-(Nitrilo­trimethyl­ene)tris­(1*H*-benzimidazol-3-ium) trinitrate

**DOI:** 10.1107/S160053681100393X

**Published:** 2011-02-12

**Authors:** Yi Cui

**Affiliations:** a School of Mechanical Engineering, Qingdao Technological University, Qingdao 266033, People’s Republic of China

## Abstract

In the title compound, C_24_H_24_N_7_^3+^·3NO_3_^−^, the cation exhibits a distorted propeller-like conformation in which the benzimid­azolium fragments form dihedral angles of 9.4 (1), 10.7 (1) and 19.1 (1)° with each other. In the crystal, inter­molecular N—H⋯O hydrogen bonds link cations and anions into double ribbons propagated in [100]. Weak inter­molecular C—H⋯O inter­actions further consolidate the packing.

## Related literature

A blue-emitting LED device fabricated with the tris­(2-amino­eth­yl)amine cerium complex was reported by Zheng *et al.* (2007 ▶). For the crystal structures of related tris(1*H*-benzimidazol-2-ylmethyl)amine adducts with H_2_O and 1.5H_2_O·0.5MeOH·MeCN, see: Zhang *et al.* (2005 ▶). 
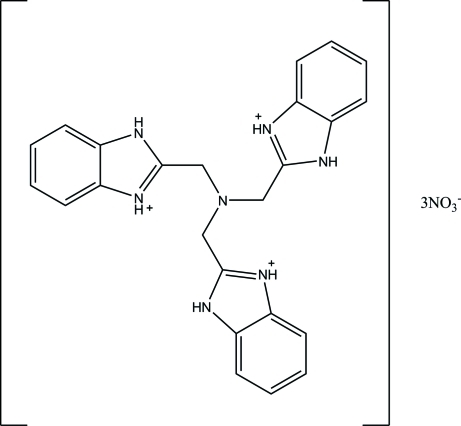


## Experimental

### 

#### Crystal data

C_24_H_24_N_7_^3+^·3NO_3_^−^
*M* *_r_* = 596.53
Triclinic, 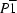

*a* = 8.9493 (3) Å
*b* = 9.2209 (3) Å
*c* = 15.8027 (6) Å
α = 98.438 (1)°
β = 91.910 (1)°
γ = 101.156 (1)°
*V* = 1263.00 (8) Å^3^
*Z* = 2
Mo *K*α radiation
μ = 0.12 mm^−1^
*T* = 153 K
0.10 × 0.10 × 0.10 mm

#### Data collection

Bruker SMART 1K CCD diffractometer
Absorption correction: multi-scan (*SADABS*; Sheldrick, 2004 ▶) *T*_min_ = 0.972, *T*_max_ = 0.985
12387 measured reflections
5703 independent reflections
4738 reflections with *I* > 2σ(*I*)
*R*_int_ = 0.014

#### Refinement

*R*[*F* ^2^ > 2σ(*F* ^2^)] = 0.034
*wR*(*F* ^2^) = 0.103
*S* = 1.09
5703 reflections
413 parameters
6 restraints
H atoms treated by a mixture of independent and constrained refinement
Δρ_max_ = 0.36 e Å^−3^
Δρ_min_ = −0.22 e Å^−3^

### 

Data collection: *SMART* (Bruker, 2001 ▶); cell refinement: *SAINT* (Bruker, 2001 ▶); data reduction: *SAINT*; program(s) used to solve structure: *SHELXTL* (Sheldrick, 2008 ▶); program(s) used to refine structure: *SHELXTL*; molecular graphics: *SHELXTL*; software used to prepare material for publication: *SHELXTL* and local programs.

## Supplementary Material

Crystal structure: contains datablocks global, I. DOI: 10.1107/S160053681100393X/cv5044sup1.cif

Structure factors: contains datablocks I. DOI: 10.1107/S160053681100393X/cv5044Isup2.hkl

Additional supplementary materials:  crystallographic information; 3D view; checkCIF report

## Figures and Tables

**Table 1:** Hydrogen-bond geometry (Å, °)

*D*—H⋯*A*	*D*—H	H⋯*A*	*D*⋯*A*	*D*—H⋯*A*
N1—H1*N*⋯O4^i^	0.87 (2)	1.95 (2)	2.8147 (15)	176 (2)
N2—H2*N*⋯O8	0.87 (2)	1.94 (2)	2.7891 (15)	167 (2)
N3—H3*N*⋯O4^i^	0.88 (1)	1.87 (1)	2.7382 (15)	172 (2)
N2—H2*N*⋯O9	0.87 (2)	2.56 (2)	3.2396 (16)	136 (2)
N5—H5*N*⋯O3	0.88 (2)	1.77 (2)	2.6477 (15)	175 (2)
N4—H4*N*⋯O5	0.86 (1)	2.10 (2)	2.8793 (16)	150 (2)
N4—H4*N*⋯O6	0.86 (1)	2.52 (1)	3.3127 (18)	153 (2)
C5—H5*A*⋯O9	0.95	2.54	3.2922 (18)	136
C8—H8*A*⋯O2^ii^	0.99	2.32	3.2585 (17)	159
C9—H9*B*⋯O2^ii^	0.99	2.47	3.2500 (16)	135
C13—H13*A*⋯O3^iii^	0.95	2.52	3.2240 (18)	131

Symmetry codes: (i) 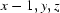; (ii) 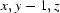; (iii) 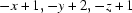.

**Fig. 1.**
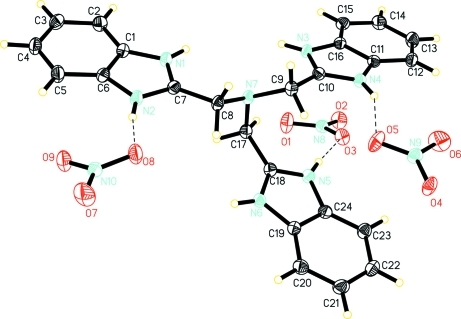
The molecular structure of (I), showing 50% probability displacement ellipsoids and the atom-numbering scheme. N—H···O hydrogen bonds are shown by dashed lines.

## supplementary crystallographic information

### Comment

The tripodal ligands derived from the Schiff-base condensation with tris(2-aminoethyl)amine (H_3_ntb) are of particular interest since the benzimidazole ring in a terpyridine-like ligand allows easy derivation and incorporation in segmental di- and trileptic ligands used as building blocks in self-assembling processes (Zheng *et al.*, 2007). A blue-emitting LED device was fabricated using one ntb cerium complex (Zheng *et al.*, 2007). In the corresponding ntb adduct [ntb.H_2_O and ntb.1.5H_2_O.0.5MeOH.MeCN] (Zhang *et al.*, 2005).the ntb adopts a tripodal 'mode to form hydrogen bonds with a solvent water molecule *via* N—H···O and O—H···N hydrogen bond. As a part of our study of the assembly of supramolecular aggregates with ntb-related compounds, we report here the synthesis and crystal structure of the title compound (I).

In (I) (Fig. 1), the three free N atoms were all pronated, and ntb adopts a tripodal mode to form strong N—H···O hydrogen bonds (Table 1) with O atoms of three nitrate anions building the 1-D chains along the *a* axis. In the cation, three benzimidazole rings form in pairs the dihedral angles in the region 9.4 (1) to 19.1 (1)°. There are also some weak C—H···O inter- and intramolecular interactions, which further stabilize the structure of (I).

### Experimental

Tris(2-aminoethyl)amine was prepared from the condensation reaction between nitrilotriacetate and 1,2-diaminobenzene in diethylene glycol (yield 73%). Single crystals of (I) suitable for X-ray analysis were obtained by slow evaporation from a 50% nitric acid solution at room temperature.

### Refinement

C-bound H atoms were geometrically positioned with C—H distances of 0.93–0.97 Å. and refined as riding, with U_iso_(H) = 1.2 U_eq_(C). N-bound H atoms were located in difference Fourier maps and refined isotropically, with N—H bond length restrained to 0.87 (2) Å.

### Crystal data

**Table:**

C_24_H_24_N_7_^3+^·3NO_3_^−^	*Z* = 2
*M**_r_* = 596.53	*F*(000) = 620
Triclinic, *P*1	*D*_x_ = 1.569 Mg m^−^^3^
Hall symbol: -P 1	Mo *K*α radiation, λ = 0.71073 Å
*a* = 8.9493 (3) Å	Cell parameters from 520 reflections
*b* = 9.2209 (3) Å	θ = 10–14°
*c* = 15.8027 (6) Å	µ = 0.12 mm^−^^1^
α = 98.438 (1)°	*T* = 153 K
β = 91.910 (1)°	Block, colorless
γ = 101.156 (1)°	0.10 × 0.10 × 0.10 mm
*V* = 1263.00 (8) Å^3^	

### Data collection

**Table:**

Bruker SMART 1K CCD diffractometer	5703 independent reflections
Radiation source: fine-focus sealed tube	4738 reflections with *I* > 2σ(*I*)
graphite	*R*_int_ = 0.014
Thin–slice ω scans	θ_max_ = 27.5°, θ_min_ = 3.1°
Absorption correction: multi-scan (*SADABS*; Sheldrick, 2004)	*h* = −11→11
*T*_min_ = 0.972, *T*_max_ = 0.985	*k* = −11→11
12387 measured reflections	*l* = −20→20

### Refinement

**Table:**

Refinement on *F*^2^	Secondary atom site location: difference Fourier map
Least-squares matrix: full	Hydrogen site location: inferred from neighbouring sites
*R*[*F*^2^ > 2σ(*F*^2^)] = 0.034	H atoms treated by a mixture of independent and constrained refinement
*wR*(*F*^2^) = 0.103	*w* = 1/[σ^2^(*F*_o_^2^) + (0.052*P*)^2^ + 0.4262*P*] where *P* = (*F*_o_^2^ + 2*F*_c_^2^)/3
*S* = 1.09	(Δ/σ)_max_ = 0.001
5703 reflections	Δρ_max_ = 0.36 e Å^−^^3^
413 parameters	Δρ_min_ = −0.22 e Å^−^^3^
6 restraints	Extinction correction: *SHELXTL* (Sheldrick, 2008), Fc^*^=kFc[1+0.001xFc^2^λ^3^/sin(2θ)]^-1/4^
Primary atom site location: structure-invariant direct methods	Extinction coefficient: 0.0084 (12)

### Special details

**Table:**

Geometry. All esds (except the esd in the dihedral angle between two l.s. planes) are estimated using the full covariance matrix. The cell esds are taken into account individually in the estimation of esds in distances, angles and torsion angles; correlations between esds in cell parameters are only used when they are defined by crystal symmetry. An approximate (isotropic) treatment of cell esds is used for estimating esds involving l.s. planes.
Refinement. Refinement of F^2^ against ALL reflections. The weighted R-factor wR and goodness of fit S are based on F^2^, conventional R-factors R are based on F, with F set to zero for negative F^2^. The threshold expression of F^2^ > 2sigma(F^2^) is used only for calculating R-factors(gt) etc. and is not relevant to the choice of reflections for refinement. R-factors based on F^2^ are statistically about twice as large as those based on F, and R- factors based on ALL data will be even larger.

### Fractional atomic coordinates and isotropic or equivalent isotropic displacement parameters (Å^2^)

**Table:**

	*x*	*y*	*z*	*U*_iso_*/*U*_eq_	
O1	0.09657 (13)	0.85873 (13)	0.28627 (8)	0.0368 (3)	
O2	0.22174 (12)	1.06093 (11)	0.36368 (7)	0.0297 (2)	
O3	0.32723 (11)	0.86872 (11)	0.33719 (6)	0.0250 (2)	
O4	0.80054 (11)	0.48938 (11)	0.36252 (7)	0.0264 (2)	
O5	0.55693 (11)	0.47164 (14)	0.36810 (8)	0.0370 (3)	
O6	0.70944 (15)	0.65439 (13)	0.44643 (8)	0.0395 (3)	
N1	−0.23863 (12)	0.23077 (12)	0.23824 (7)	0.0189 (2)	
N2	−0.17444 (12)	0.05714 (13)	0.14645 (7)	0.0211 (2)	
N3	0.09014 (12)	0.61176 (12)	0.43150 (7)	0.0176 (2)	
N4	0.33476 (12)	0.62438 (12)	0.44885 (7)	0.0174 (2)	
N5	0.36487 (13)	0.63293 (12)	0.22864 (7)	0.0195 (2)	
N6	0.31324 (13)	0.43181 (13)	0.13275 (7)	0.0198 (2)	
N7	0.07227 (12)	0.38082 (12)	0.28051 (7)	0.0170 (2)	
N8	0.21321 (13)	0.93021 (13)	0.32878 (7)	0.0203 (2)	
N9	0.68763 (13)	0.53995 (13)	0.39242 (7)	0.0225 (2)	
C1	−0.37391 (15)	0.14343 (14)	0.19857 (8)	0.0182 (3)	
C2	−0.52618 (15)	0.14841 (16)	0.21100 (9)	0.0228 (3)	
H2A	−0.5549	0.2226	0.2520	0.027*	
C3	−0.63329 (15)	0.03933 (16)	0.16042 (9)	0.0237 (3)	
H3A	−0.7386	0.0385	0.1670	0.028*	
C4	−0.59187 (16)	−0.06976 (16)	0.09986 (9)	0.0248 (3)	
H4A	−0.6695	−0.1418	0.0660	0.030*	
C5	−0.44094 (16)	−0.07550 (16)	0.08808 (9)	0.0251 (3)	
H5A	−0.4123	−0.1499	0.0472	0.030*	
C6	−0.33291 (14)	0.03357 (15)	0.13940 (8)	0.0188 (3)	
C7	−0.12222 (15)	0.17477 (14)	0.20604 (8)	0.0179 (3)	
C8	0.04170 (14)	0.22638 (14)	0.23621 (9)	0.0189 (3)	
H8A	0.0697	0.1603	0.2756	0.023*	
H8B	0.1057	0.2190	0.1865	0.023*	
C9	0.19329 (14)	0.40230 (14)	0.34832 (8)	0.0184 (3)	
H9A	0.2919	0.4008	0.3220	0.022*	
H9B	0.1729	0.3183	0.3815	0.022*	
C10	0.20520 (14)	0.54612 (14)	0.40762 (8)	0.0167 (2)	
C11	0.30374 (14)	0.74700 (14)	0.50206 (8)	0.0176 (3)	
C12	0.39816 (15)	0.86314 (15)	0.55629 (9)	0.0218 (3)	
H12A	0.5049	0.8685	0.5640	0.026*	
C13	0.32688 (17)	0.97029 (16)	0.59821 (9)	0.0257 (3)	
H13A	0.3869	1.0523	0.6358	0.031*	
C14	0.16882 (16)	0.96283 (16)	0.58755 (9)	0.0244 (3)	
H14A	0.1253	1.0392	0.6183	0.029*	
C15	0.07526 (15)	0.84738 (15)	0.53349 (8)	0.0208 (3)	
H15A	−0.0315	0.8421	0.5259	0.025*	
C16	0.14675 (14)	0.73898 (14)	0.49072 (8)	0.0173 (3)	
C17	0.09618 (14)	0.49279 (14)	0.22158 (8)	0.0185 (3)	
H17A	0.0746	0.5882	0.2511	0.022*	
H17B	0.0227	0.4578	0.1711	0.022*	
C18	0.25514 (15)	0.52073 (14)	0.19152 (8)	0.0181 (3)	
C19	0.46906 (15)	0.48775 (15)	0.13232 (8)	0.0192 (3)	
C20	0.58288 (16)	0.43787 (16)	0.08460 (9)	0.0237 (3)	
H20A	0.5605	0.3513	0.0419	0.028*	
C21	0.72911 (16)	0.52014 (17)	0.10235 (9)	0.0250 (3)	
H21A	0.8094	0.4899	0.0708	0.030*	
C22	0.76262 (16)	0.64709 (16)	0.16559 (9)	0.0247 (3)	
H22A	0.8653	0.6999	0.1765	0.030*	
C23	0.65013 (16)	0.69746 (15)	0.21250 (9)	0.0228 (3)	
H23A	0.6728	0.7837	0.2554	0.027*	
C24	0.50187 (15)	0.61558 (14)	0.19387 (8)	0.0190 (3)	
H4N	0.4215 (13)	0.6001 (19)	0.4400 (11)	0.032 (5)*	
H1N	−0.227 (2)	0.3081 (15)	0.2784 (10)	0.041 (5)*	
H2N	−0.121 (2)	0.0014 (19)	0.1165 (11)	0.043 (5)*	
H3N	−0.0046 (12)	0.580 (2)	0.4113 (12)	0.041 (5)*	
H5N	0.346 (2)	0.7090 (16)	0.2639 (11)	0.047 (6)*	
H6N	0.263 (2)	0.3528 (16)	0.0987 (11)	0.043 (5)*	
O7	0.02490 (14)	−0.30337 (14)	−0.02717 (8)	0.0398 (3)	
O8	0.02242 (11)	−0.12078 (12)	0.07496 (7)	0.0311 (3)	
O9	−0.16701 (12)	−0.19196 (12)	−0.01998 (7)	0.0287 (2)	
N10	−0.03802 (13)	−0.20607 (13)	0.00941 (7)	0.0221 (2)	

### Atomic displacement parameters (Å^2^)

**Table:**

	*U*^11^	*U*^22^	*U*^33^	*U*^12^	*U*^13^	*U*^23^
O1	0.0259 (5)	0.0319 (6)	0.0470 (7)	0.0034 (5)	−0.0091 (5)	−0.0065 (5)
O2	0.0341 (6)	0.0190 (5)	0.0359 (6)	0.0113 (4)	0.0015 (5)	−0.0041 (4)
O3	0.0276 (5)	0.0216 (5)	0.0270 (5)	0.0127 (4)	−0.0016 (4)	−0.0011 (4)
O4	0.0135 (4)	0.0282 (5)	0.0353 (6)	0.0080 (4)	0.0015 (4)	−0.0072 (4)
O5	0.0134 (5)	0.0479 (7)	0.0496 (7)	0.0061 (5)	−0.0002 (4)	0.0077 (6)
O6	0.0485 (7)	0.0303 (6)	0.0397 (7)	0.0162 (5)	0.0092 (5)	−0.0071 (5)
N1	0.0154 (5)	0.0180 (5)	0.0216 (5)	0.0031 (4)	0.0003 (4)	−0.0015 (4)
N2	0.0153 (5)	0.0207 (6)	0.0248 (6)	0.0033 (4)	0.0014 (4)	−0.0043 (5)
N3	0.0137 (5)	0.0188 (5)	0.0194 (5)	0.0035 (4)	0.0002 (4)	0.0001 (4)
N4	0.0140 (5)	0.0185 (5)	0.0198 (5)	0.0056 (4)	0.0007 (4)	0.0000 (4)
N5	0.0198 (5)	0.0162 (5)	0.0208 (5)	0.0030 (4)	0.0018 (4)	−0.0015 (4)
N6	0.0175 (5)	0.0204 (6)	0.0196 (5)	0.0036 (5)	0.0005 (4)	−0.0028 (4)
N7	0.0136 (5)	0.0157 (5)	0.0205 (5)	0.0034 (4)	−0.0003 (4)	−0.0014 (4)
N8	0.0216 (6)	0.0196 (5)	0.0202 (5)	0.0060 (5)	0.0023 (4)	0.0022 (4)
N9	0.0190 (5)	0.0256 (6)	0.0250 (6)	0.0085 (5)	0.0036 (4)	0.0044 (5)
C1	0.0172 (6)	0.0166 (6)	0.0203 (6)	0.0026 (5)	−0.0005 (5)	0.0022 (5)
C2	0.0192 (6)	0.0239 (7)	0.0257 (7)	0.0076 (5)	0.0017 (5)	0.0010 (5)
C3	0.0150 (6)	0.0273 (7)	0.0296 (7)	0.0050 (5)	0.0009 (5)	0.0063 (6)
C4	0.0189 (6)	0.0263 (7)	0.0260 (7)	0.0000 (6)	−0.0046 (5)	0.0008 (6)
C5	0.0205 (6)	0.0248 (7)	0.0259 (7)	0.0019 (6)	−0.0010 (5)	−0.0050 (5)
C6	0.0151 (6)	0.0196 (6)	0.0215 (6)	0.0034 (5)	0.0005 (5)	0.0030 (5)
C7	0.0171 (6)	0.0159 (6)	0.0199 (6)	0.0029 (5)	0.0020 (5)	0.0010 (5)
C8	0.0140 (6)	0.0173 (6)	0.0236 (6)	0.0034 (5)	0.0000 (5)	−0.0026 (5)
C9	0.0169 (6)	0.0167 (6)	0.0210 (6)	0.0054 (5)	−0.0005 (5)	−0.0010 (5)
C10	0.0160 (6)	0.0179 (6)	0.0168 (6)	0.0049 (5)	0.0006 (5)	0.0026 (5)
C11	0.0175 (6)	0.0191 (6)	0.0163 (6)	0.0043 (5)	0.0019 (5)	0.0021 (5)
C12	0.0177 (6)	0.0235 (7)	0.0218 (6)	0.0019 (5)	−0.0005 (5)	−0.0008 (5)
C13	0.0253 (7)	0.0232 (7)	0.0246 (7)	0.0026 (6)	−0.0013 (5)	−0.0056 (5)
C14	0.0267 (7)	0.0230 (7)	0.0232 (7)	0.0092 (6)	0.0035 (5)	−0.0027 (5)
C15	0.0188 (6)	0.0230 (7)	0.0218 (6)	0.0073 (5)	0.0041 (5)	0.0025 (5)
C16	0.0167 (6)	0.0179 (6)	0.0170 (6)	0.0031 (5)	0.0015 (5)	0.0019 (5)
C17	0.0161 (6)	0.0179 (6)	0.0210 (6)	0.0052 (5)	0.0000 (5)	−0.0007 (5)
C18	0.0184 (6)	0.0174 (6)	0.0185 (6)	0.0043 (5)	0.0000 (5)	0.0018 (5)
C19	0.0176 (6)	0.0203 (6)	0.0193 (6)	0.0031 (5)	0.0006 (5)	0.0023 (5)
C20	0.0240 (7)	0.0245 (7)	0.0219 (6)	0.0073 (6)	0.0027 (5)	−0.0020 (5)
C21	0.0212 (7)	0.0308 (7)	0.0252 (7)	0.0093 (6)	0.0046 (5)	0.0056 (6)
C22	0.0172 (6)	0.0270 (7)	0.0297 (7)	0.0023 (6)	0.0007 (5)	0.0062 (6)
C23	0.0209 (6)	0.0199 (6)	0.0263 (7)	0.0027 (5)	0.0007 (5)	0.0008 (5)
C24	0.0203 (6)	0.0185 (6)	0.0186 (6)	0.0052 (5)	0.0019 (5)	0.0027 (5)
O7	0.0381 (6)	0.0415 (7)	0.0384 (6)	0.0190 (5)	0.0028 (5)	−0.0135 (5)
O8	0.0211 (5)	0.0365 (6)	0.0292 (5)	0.0039 (4)	−0.0023 (4)	−0.0127 (4)
O9	0.0237 (5)	0.0295 (5)	0.0298 (5)	0.0071 (4)	−0.0061 (4)	−0.0058 (4)
N10	0.0201 (5)	0.0225 (6)	0.0213 (6)	0.0022 (5)	0.0042 (4)	−0.0026 (4)

### Geometric parameters (Å, °)

**Table:**

O1—N8	1.2380 (16)	C5—C6	1.3914 (18)
O2—N8	1.2367 (15)	C5—H5A	0.9500
O3—N8	1.2717 (15)	C7—C8	1.4908 (17)
O4—N9	1.2694 (14)	C8—H8A	0.9900
O5—N9	1.2355 (16)	C8—H8B	0.9900
O6—N9	1.2349 (16)	C9—C10	1.4907 (17)
N1—C7	1.3332 (16)	C9—H9A	0.9900
N1—C1	1.3897 (16)	C9—H9B	0.9900
N1—H1N	0.869 (9)	C11—C12	1.3883 (18)
N2—C7	1.3254 (17)	C11—C16	1.3966 (18)
N2—C6	1.3907 (16)	C12—C13	1.379 (2)
N2—H2N	0.869 (9)	C12—H12A	0.9500
N3—C10	1.3301 (16)	C13—C14	1.406 (2)
N3—C16	1.3874 (16)	C13—H13A	0.9500
N3—H3N	0.874 (9)	C14—C15	1.3801 (19)
N4—C10	1.3333 (16)	C14—H14A	0.9500
N4—C11	1.3859 (16)	C15—C16	1.3940 (18)
N4—H4N	0.859 (9)	C15—H15A	0.9500
N5—C18	1.3301 (17)	C17—C18	1.5016 (17)
N5—C24	1.3877 (17)	C17—H17A	0.9900
N5—H5N	0.876 (9)	C17—H17B	0.9900
N6—C18	1.3338 (17)	C19—C24	1.3900 (18)
N6—C19	1.3906 (17)	C19—C20	1.3959 (18)
N6—H6N	0.873 (9)	C20—C21	1.377 (2)
N7—C8	1.4618 (16)	C20—H20A	0.9500
N7—C9	1.4621 (16)	C21—C22	1.400 (2)
N7—C17	1.4795 (16)	C21—H21A	0.9500
C1—C6	1.3870 (18)	C22—C23	1.381 (2)
C1—C2	1.3918 (18)	C22—H22A	0.9500
C2—C3	1.382 (2)	C23—C24	1.3923 (19)
C2—H2A	0.9500	C23—H23A	0.9500
C3—C4	1.397 (2)	O7—N10	1.2334 (16)
C3—H3A	0.9500	O8—N10	1.2422 (15)
C4—C5	1.3796 (19)	O9—N10	1.2671 (15)
C4—H4A	0.9500		
			
C7—N1—C1	108.62 (11)	C10—C9—H9A	109.1
C7—N1—H1N	123.1 (13)	N7—C9—H9B	109.1
C1—N1—H1N	128.3 (13)	C10—C9—H9B	109.1
C7—N2—C6	108.74 (11)	H9A—C9—H9B	107.9
C7—N2—H2N	127.1 (13)	N3—C10—N4	109.55 (11)
C6—N2—H2N	124.1 (13)	N3—C10—C9	126.25 (11)
C10—N3—C16	108.76 (11)	N4—C10—C9	124.10 (11)
C10—N3—H3N	124.8 (13)	N4—C11—C12	131.68 (12)
C16—N3—H3N	126.5 (13)	N4—C11—C16	106.07 (11)
C10—N4—C11	109.06 (11)	C12—C11—C16	122.23 (12)
C10—N4—H4N	122.4 (12)	C13—C12—C11	115.64 (13)
C11—N4—H4N	128.5 (12)	C13—C12—H12A	122.2
C18—N5—C24	108.72 (11)	C11—C12—H12A	122.2
C18—N5—H5N	122.6 (14)	C12—C13—C14	122.55 (13)
C24—N5—H5N	128.2 (14)	C12—C13—H13A	118.7
C18—N6—C19	108.57 (11)	C14—C13—H13A	118.7
C18—N6—H6N	126.8 (13)	C15—C14—C13	121.68 (13)
C19—N6—H6N	124.7 (13)	C15—C14—H14A	119.2
C8—N7—C9	110.26 (10)	C13—C14—H14A	119.2
C8—N7—C17	113.30 (10)	C14—C15—C16	116.02 (12)
C9—N7—C17	114.46 (10)	C14—C15—H15A	122.0
O2—N8—O1	121.43 (12)	C16—C15—H15A	122.0
O2—N8—O3	118.84 (11)	N3—C16—C15	131.56 (12)
O1—N8—O3	119.73 (11)	N3—C16—C11	106.56 (11)
O6—N9—O5	120.93 (12)	C15—C16—C11	121.87 (12)
O6—N9—O4	119.88 (12)	N7—C17—C18	113.13 (10)
O5—N9—O4	119.17 (12)	N7—C17—H17A	109.0
C6—C1—N1	106.39 (11)	C18—C17—H17A	109.0
C6—C1—C2	121.54 (12)	N7—C17—H17B	109.0
N1—C1—C2	132.03 (12)	C18—C17—H17B	109.0
C3—C2—C1	116.25 (13)	H17A—C17—H17B	107.8
C3—C2—H2A	121.9	N5—C18—N6	109.73 (11)
C1—C2—H2A	121.9	N5—C18—C17	123.04 (12)
C2—C3—C4	122.15 (13)	N6—C18—C17	126.80 (12)
C2—C3—H3A	118.9	C24—C19—N6	106.41 (11)
C4—C3—H3A	118.9	C24—C19—C20	121.45 (12)
C5—C4—C3	121.58 (13)	N6—C19—C20	132.15 (12)
C5—C4—H4A	119.2	C21—C20—C19	116.78 (13)
C3—C4—H4A	119.2	C21—C20—H20A	121.6
C4—C5—C6	116.35 (13)	C19—C20—H20A	121.6
C4—C5—H5A	121.8	C20—C21—C22	121.76 (13)
C6—C5—H5A	121.8	C20—C21—H21A	119.1
C1—C6—N2	106.50 (11)	C22—C21—H21A	119.1
C1—C6—C5	122.10 (12)	C23—C22—C21	121.64 (13)
N2—C6—C5	131.38 (13)	C23—C22—H22A	119.2
N2—C7—N1	109.74 (11)	C21—C22—H22A	119.2
N2—C7—C8	124.00 (11)	C22—C23—C24	116.70 (13)
N1—C7—C8	126.08 (11)	C22—C23—H23A	121.7
N7—C8—C7	111.44 (10)	C24—C23—H23A	121.7
N7—C8—H8A	109.3	N5—C24—C19	106.57 (11)
C7—C8—H8A	109.3	N5—C24—C23	131.74 (12)
N7—C8—H8B	109.3	C19—C24—C23	121.66 (12)
C7—C8—H8B	109.3	O7—N10—O8	121.37 (12)
H8A—C8—H8B	108.0	O7—N10—O9	119.80 (12)
N7—C9—C10	112.30 (10)	O8—N10—O9	118.84 (11)
N7—C9—H9A	109.1		
			
C7—N1—C1—C6	−1.23 (14)	C11—C12—C13—C14	0.4 (2)
C7—N1—C1—C2	176.57 (14)	C12—C13—C14—C15	−0.5 (2)
C6—C1—C2—C3	−0.8 (2)	C13—C14—C15—C16	0.3 (2)
N1—C1—C2—C3	−178.35 (13)	C10—N3—C16—C15	178.43 (13)
C1—C2—C3—C4	−0.2 (2)	C10—N3—C16—C11	−0.29 (14)
C2—C3—C4—C5	0.8 (2)	C14—C15—C16—N3	−178.53 (13)
C3—C4—C5—C6	−0.3 (2)	C14—C15—C16—C11	0.03 (19)
N1—C1—C6—N2	0.92 (14)	N4—C11—C16—N3	0.22 (13)
C2—C1—C6—N2	−177.16 (12)	C12—C11—C16—N3	178.73 (12)
N1—C1—C6—C5	179.44 (12)	N4—C11—C16—C15	−178.66 (12)
C2—C1—C6—C5	1.4 (2)	C12—C11—C16—C15	−0.1 (2)
C7—N2—C6—C1	−0.30 (15)	C8—N7—C17—C18	−81.34 (13)
C7—N2—C6—C5	−178.63 (14)	C9—N7—C17—C18	46.25 (14)
C4—C5—C6—C1	−0.7 (2)	C24—N5—C18—N6	−1.14 (15)
C4—C5—C6—N2	177.38 (14)	C24—N5—C18—C17	171.75 (12)
C6—N2—C7—N1	−0.48 (15)	C19—N6—C18—N5	0.82 (15)
C6—N2—C7—C8	174.92 (12)	C19—N6—C18—C17	−171.74 (12)
C1—N1—C7—N2	1.07 (15)	N7—C17—C18—N5	−95.69 (14)
C1—N1—C7—C8	−174.21 (12)	N7—C17—C18—N6	75.95 (17)
C9—N7—C8—C7	147.42 (11)	C18—N6—C19—C24	−0.18 (14)
C17—N7—C8—C7	−82.83 (13)	C18—N6—C19—C20	179.29 (14)
N2—C7—C8—N7	162.01 (12)	C24—C19—C20—C21	0.9 (2)
N1—C7—C8—N7	−23.35 (18)	N6—C19—C20—C21	−178.50 (14)
C8—N7—C9—C10	−167.25 (10)	C19—C20—C21—C22	0.5 (2)
C17—N7—C9—C10	63.62 (13)	C20—C21—C22—C23	−1.0 (2)
C16—N3—C10—N4	0.25 (14)	C21—C22—C23—C24	0.1 (2)
C16—N3—C10—C9	176.69 (12)	C18—N5—C24—C19	1.00 (15)
C11—N4—C10—N3	−0.11 (14)	C18—N5—C24—C23	−176.96 (14)
C11—N4—C10—C9	−176.64 (11)	N6—C19—C24—N5	−0.49 (14)
N7—C9—C10—N3	34.37 (17)	C20—C19—C24—N5	179.97 (12)
N7—C9—C10—N4	−149.68 (12)	N6—C19—C24—C23	177.72 (12)
C10—N4—C11—C12	−178.39 (13)	C20—C19—C24—C23	−1.8 (2)
C10—N4—C11—C16	−0.07 (14)	C22—C23—C24—N5	178.97 (14)
N4—C11—C12—C13	178.02 (13)	C22—C23—C24—C19	1.3 (2)
C16—C11—C12—C13	−0.07 (19)		

### Hydrogen-bond geometry (Å, °)

**Table:**

*D*—H···*A*	*D*—H	H···*A*	*D*···*A*	*D*—H···*A*
N1—H1N···O4^i^	0.87 (2)	1.95 (2)	2.8147 (15)	176.(2)
N2—H2N···O8	0.87 (2)	1.94 (2)	2.7891 (15)	167.(2)
N3—H3N···O4^i^	0.88 (1)	1.87 (1)	2.7382 (15)	172.(2)
N2—H2N···O9	0.87 (2)	2.56 (2)	3.2396 (16)	136.(2)
N5—H5N···O3	0.88 (2)	1.77 (2)	2.6477 (15)	175.(2)
N4—H4N···O5	0.86 (1)	2.10 (2)	2.8793 (16)	150.(2)
N4—H4N···O6	0.86 (1)	2.52 (1)	3.3127 (18)	153.(2)
C5—H5A···O9	0.95	2.54	3.2922 (18)	136
C8—H8A···O2^ii^	0.99	2.32	3.2585 (17)	159
C9—H9B···O2^ii^	0.99	2.47	3.2500 (16)	135
C13—H13A···O3^iii^	0.95	2.52	3.2240 (18)	131

Symmetry codes: (i) *x*−1, *y*, *z*; (ii) *x*, *y*−1, *z*; (iii) −*x*+1, −*y*+2, −*z*+1.
